# Identification of Tumor Antigen AF20 as Glycosylated Transferrin Receptor 1 in Complex with Heat Shock Protein 90 and/or Transporting ATPase

**DOI:** 10.1371/journal.pone.0165227

**Published:** 2016-11-01

**Authors:** Jason M. Shapiro, Waihong Chung, Kosuke Ogawa, Luke Barker, Rolf Carlson, Jack R. Wands, Jisu Li

**Affiliations:** 1 Liver Research Center, Rhode Island Hospital and Warren Alpert Medical School of Brown University, Providence, Rhode Island, United States of America; 2 Division of Pediatric Gastroenterology, Nutrition and Liver Diseases, Hasbro Children’s Hospital, Providence, Rhode Island, United States of America; Academia Sinica, TAIWAN

## Abstract

We previously isolated AF20, a murine monoclonal antibody that recognizes a cell surface glycoprotein of approximately 90–110 kDa. The AF20 antigen is specifically expressed in human hepatoma and colon cancer cell lines, and thus could serve as a cancer biomarker. To uncover the molecular identity of the AF20 antigen, a combination of ion-exchange chromatography, immunoprecipitation, and SDS—polyacrylamide gel electrophoresis was employed to purify the AF20 antigen followed by trypsin digestion and mass spectrometry. Surprisingly, three host proteins were thus purified from human hepatoma and colon cancer cell lines: transferrin receptor 1 (TFR1), heat shock protein 90 (HSP90), and Na^+^/K^+^ ATPase or Mg^++^ ATPase. Co-immunoprecipitation followed by Western blot analysis confirmed interaction among the three proteins. However, only the cDNA encoding TFR1 conferred strong cell surface staining by the AF20 antibody following its transient transfection into a cell line lacking endogenous AF20. In support of the molecular identity of AF20 as TFR1, diferric but not iron-free transferrin could prevent AF20 antigen-antibody interaction during immunoprecipitation. Moreover, very similar patterns of AF20 and TFR1 overexpression was documented in colon cancer tissues. In conclusion, AF20 is glycosylated TFR1. This finding could explain the molecular structure of AF20, its cell surface localization, as well as overexpression in cancer cells. Glycosylated TFR1 should serve as a usefulness target for anti-cancer therapy, or a vehicle for delivery of anti-tumor drugs with high affinity and specificity. The biological significance of the complex formation between TFR1, HSP90, and/or transporting ATPase warrants further investigation.

## Introduction

Hepatocellular carcinoma (HCC) is one of the most devastating cancers in the world. It is highly chemoresistant with no effective therapies. Early detection is crucial for timely treatment as well as for prevention of cancer progression and metastasis, so as to reduce morbidity and mortality. Currently, detection of pre-cancerous lesions or the early stage of HCC remains challenging. There is an urgent need to develop reliable cancer biomarkers, which may serve as therapeutic targets as well.

By immunizing mice with hepatoma cells derived from an HCC cell line (FOCUS), we previously isolated a handful of monoclonal antibodies with high affinity to cancer cells but not to normal or non-transformed hepatocytes [1]. One of the antibodies, namely AF20, recognized a protein of apparent molecular size of 90–110 kDa. The AF20 antigen was found abundantly expressed on cell surface of human HCC cell lines, as well as human colon cancer cell lines such as LS180 and HT-29 [2,3]. Binding of AF20 antibody to the cancer cells was followed by its rapid internalization, which raises the hope of specific and highly efficient delivery of therapeutic drugs or small molecules into cancer cells [3,4]. *In vivo* experiments validated AF20’s ability to differentiate human HCC from adjacent normal liver tissue [1]. Therefore, AF20 may serve as a potential biomarker for early detection and diagnosis of malignant transformation, and also as a vehicle for delivery of anti-tumor drugs with high affinity and specificity. Further characterization revealed AF20 antigen as a dimer of 90-kDa glycoprotein linked together by disulfide bonds [3]. In the present study, we demonstrated that AF20 antigen is identical to the glycosylated form of human transferrin receptor 1 (TRF1), which can form a protein complex with heat shock protein 90 (HSP90) and/or transporting ATPase.

## Materials and Methods

### Plasmids

Expression constructs for TFR1 (Myc-DDK-tagged, RC200980, NM_003234.1), HSP90 (untagged, SC108085, NM_007355.2), and Na^+^/K^+^ ATPase (Myc-DDK-tagged, RC201009, NM_000701) were purchased from Origene.

### Antibodies and reagents

AF20 monoclonal antibody (mAb) was produced and characterized as previously described [1,3,4]. The following antibodies were obtained commercially: anti-TFR1 (CD71) (Santa Cruz Biotechnology, Inc.; sc-32272), anti-HSP90 (EMD Millipore; 05–594), anti-N^+^/K^+^ ATPase (abcam, ab7671) and anti-DDK (Origene; TA100011). PNGase F was purchased from New England Biolabs (P0704S). Apo transferrin and holo transferrin were purchased from EMB Millipore (616395 and 616397).

### Cell culture and transfection

Human hepatoma cell line Huh7 (our lab stock), human kidney cell line BOSC (ATCC), and monkey kidney cell line COS-1 (ATCC) were cultured in Dulbecco’s Modified Eagle Medium (DMEM) supplemented with 10% fetal bovine serum (FBS). HepG2 (human hepatoma, ATCC), LS180 (human colon cancer, ATCC), and NIH 3T3 (mouse fibroblast, ATCC) cells were cultured in EMEM supplemented with 10% FBS and 1% non-essential amino acids. HepaRG (hepatic cell line, from Dr. Christian Trepo, Lyon, France) was cultured in William’s E medium supplemented with 10% FBS, bovine insulin (5μg/ml) and 7x10^-5^ M hydrocortisone. Cells grown in 6-well or 24-well plates with cover slips were transfected with 1μg or 0.25μg of TFR1 or other cDNA expression constructs using polyamine as a carrier (Mirus), and harvested 2 days later for Western blot analysis or fixed for immunofluorescent (IF) staining.

### Purification of AF20 antigen and protein identification

Two grams of cell pellet was resuspended in water supplemented with a protease cocktail, followed by four cycles of freezing/thawing in dry ice/methanol bath and 37°C water bath, respectively. Ten times concentrated phosphate buffered saline (PBS) was added to a final concentration of 1x. After centrifugation at 14,000 rpm for 10 min, cell lysate was mixed with 1M Tris-HCL, pH 8.0 to a final concentration of 50mM, and loaded onto a column containing 20ml DEAE-cellulose. After flow-through, the column was washed three times with 20ml each of 50mM Tris pH 8.0. Proteins bound to DEAE cellulose column were eluted successively with 100mM, 200mM, and 400mM NaCl prepared in 50mM Tris, pH 8.0. Each fraction was collected and BCA assay was performed to measure protein concentration. Protein peaks were collected and subject to immunoprecipitation (IP) using AF20 antibody followed by Western blot with the same antibody. Fractions containing AF20 antigen were pooled for large scale IP followed by separation in 10% SDS-polyacrylamide gel (PAGE). Protein bands(s) corresponding to the size of AF20 (90-110kDa) were excised and subject to trypsin digestion, followed by peptide separation and mass spectrometry (MS) analysis on a LTQ Orbitrap mass spectrometer at Keck Biotechnology Resource Laboratory, Yale University.

### IP-Western blot analysis

Cell lysate was incubated at 4°C overnight with AF20 antibody immobilized on protein G beads. After washing with PBS, bound proteins were separated in 8% or 10% SDS-PAGE, transferred to polyvinylidene fluoride (PVDF) membrane, blocked with 3% milk in PBS supplemented with 0.05% Tween 20 (PBST), and incubated with AF20 antibody overnight in the same solution. After washing with PBS, the membrane was incubated at room temperature (RT) for 1hr with anti-mouse antibody conjugated with horseradish peroxidase (HRP). Signals were revealed by enhanced chemiluminescence (ECL) followed by exposure to X-ray film.

### IF staining

Cells grown on cover slips were fixed at -20°C with acid ethanol (5% acetic acid glacial, 95% ethanol) for at least 2 hrs. IF staining was performed using Immnostaining kit (Active Motif, 15251). Briefly, after washing with PBS and Maxwash, fixed cells were incubated successively, at 4°C for 1hr, with Maxblock, primary antibody in Maxbind, and secondary antibody in Maxbind. After washing with Maxwash, the cover slips were transferred onto glass slide using mounting solution containing DAPI, and signals were examined under UV microscope.

### Protein deglycosylation

Huh7 cell lysate was subject to IP with AF20 antibody as described above. After washing with PBS, bound proteins were incubated with PNGase F at 37°C for at least 1 hr under conditions recommended by the Manufacturer. Samples without addition of the enzyme were incubated in parallel for controls. Next, samples were loaded to 10% SDS-PAGE for Western blot analysis using AF20 and TFR1 mAbs, respectively.

### Immunohistochemistry

Formalin fixed, paraffin-embedded sections were deparaffinized by xylene followed by an ethanol gradient. Antigen retrieval was done by microwaving deparaffinized sections submerged in preheated Antigen Unmasking Solution H-3300 (Vector Laboratories, California) in a pressure cooker (Nordic Ware, Minnesota) for 2 minutes. Sections were cooled to room temperature in a water bath and then submerged in an endogenous peroxidase blocking solution containing 3% H_2_O_2_ in methanol for 30 minutes. The rest of the staining process, including blocking, primary and secondary antibody incubation, and peroxidase-labeling, was performed using the Elite ABC Kit PK-6102 (Vector Laboratories, California) according to manufacturer's instruction. Primary antibody was diluted 1:50–1:500 and incubated at 4°C overnight. Sections were washed in PBST between each incubation step. Color development was done using DAB Peroxidase Substrate SK-4103 (Vector Laboratories, California). Developed sections were counterstained by hematoxylin. Finally, sections were dehydrated using a reversed ethanol gradient followed by xylene.

## Results

### Comparison of AF20 protein levels among cell lines of diverse origins

**Fig 1:**
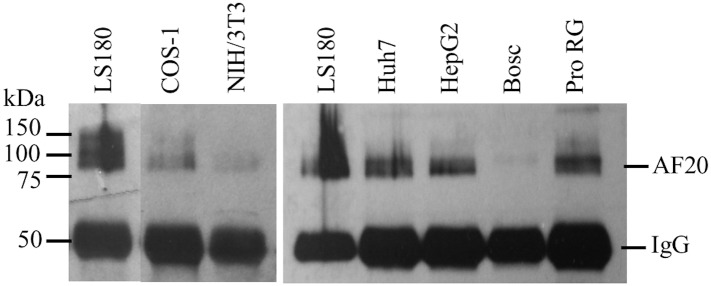
Comparison of AF20 antigen levels in different cell lines. Cell lysate containing 200μg of proteins was subject to immunoprecipitation with AF20 antibody followed by Western blot using the same antibody. The 50-kDd protein band in the blot corresponds to the heavy chain of AF20 antibody (IgG). Pro RG: proliferating HepaRG cells. Note that for the left panel, lane 1 and lanes 2/3 were cropped from different parts of the same blot.

We previously demonstrated robust AF20 expression in hepatoma and colon cancer cell lines [1,3]. IF staining indicated that AF20 antigen is localized on cell surface of hepatoma cells. Quantitative analysis revealed highest levels of AF20 antigen in hepatoma cell lines such as FOCUS and Huh7, as well as LS180, a colon cancer cell line. In contrast, NIH 3T3 and COS-1 cells showed no or little AF20 expression [3]. To compare expression level of AF20 in cancer cells of diverse origins, we performed quantitative IP-Western blot analysis. AF20 expression was high in LS180, Huh7, HepG2, and proliferating HepaRG cells, a liver stem cell line, but low in Bosc, Cos-1 (human and monkey kidney), and NIH 3T3 (mouse embryonic fibroblast) cells (Fig 1). These findings are highly consistent with our previous reports.

### AF20 mAb immunoprecipitated TFR1 and other host proteins

**Fig 2:**
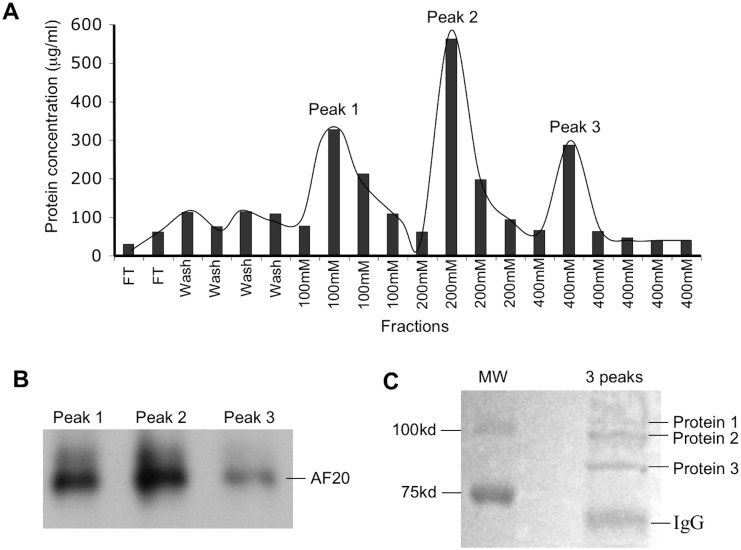
Purification of AF20 antigen from Huh7 cells for proteomic analysis. **A**. Cell pellet (2 grams) was lyzed by freeze/thaw and diluted with Tris buffer. The sample was loaded onto a DEAE-cellulose column. After flow through (FT), the column was washed with Tris buffer (wash). Proteins were eluted successively with 100 mM, 200 mM and 400 mM of NaCl, and protein concentration was determined by BCA assay using known concentrations of albumin as a standard. **(B & C)** AF20 antigen was imunoprecipitated from the three protein peaks by AF20 mAb and subjected to Western blot (minigel format) using the same antibody (B), or pooled and subject to SDS-PAGE (large gel format) followed by Coomassie blue staining (C). The area of three protein bands was cut out for proteomic analysis. MW: molecular size markers.

A combination of ion-exchange chromatography and affinity chromatography was used to purify AF20 protein from Huh7 cells. The cell lysate was loaded onto DEAE-cellulose column, and bound proteins were eluted sequentially with 100, 200, and 400mM NaCl solution (Fig 2A). Surprisingly, IP-Western blot analysis revealed presence of AF20 antigen in protein peaks from all the three NaCl concentrations (Fig 2B). Protein peaks of the three NaCl concentrations were pooled for concentration by Centricon-30. After separation in SDS-PAGE, the large gel was stained with Coomassie blue. Three protein bands in the range of 90 to 110 kDd were visible (Fig 2C). They were cut out and subject to mass spectrometry as detailed in Materials and Methods. The results, as shown in Table 1, revealed presence of three proteins of similar molecular weights from Huh7 cells: transferrin receptor 1 (TFR1), heat shock protein 90 (HSP90), and Na^+^/K^+^ ATPase or Mg^++^ ATPase. Using each of the three bands for proteomic analysis still revealed presence of all the three proteins. Similar results were obtained using cell lysate from FOCUS, LS180, and HepG2 cells, although ATPase was not always present (Table 1 and data not shown). It should be pointed out that we did not attempt to use proteins from a single NaCl concentration for proteomic analysis.

**Table 1:** Protein sequencing results.

Cell line	Protein identity	ID	MW
*FOCUS/LS180			
180 Kda:	TFR1	gi37433	84848
90–100 Kda:	Na^+^/K^+^ ATPase	gi21361181	112842
	HSP gp96 precursor	gi15010550	90138
	TFR1	gi37433	84848
	HSP 90-β	gi20149594	83212
	HSP 90-α2	gi61656603	98052
Huh7	Mg^++^ATPase (Pseudomonas)	gi395497517	99809
	HSP 90	gi306891	83242
	HSP 90-α	gi12082136	83046
LS180	TFR1	P02786	84818
	HSP 90-α	P07900	84607
HepG2	TFR1	P02786	84818
	HSP 90-β	P08238	83212
	Na^+^/K^+^ ATPase subunit α1	P05023	112824
	HSP 90-α	P07900	84607
FOCUS	TFR1	P02786	84818
	HSP 90-β	P08238	83212

*Mixed lysate from FOCUS and LS180 cells

### Validation of TFR1 as an AF20 antigen

**Fig 3:**
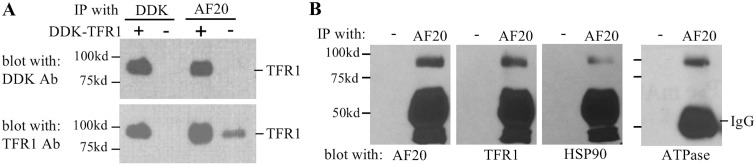
AF20 antibody recognizes TFR1 but could co-immunoprecipitate HSP90 and Na^+^/K^+^ ATPase from Huh7 cells. **A**. Huh7 cells were transiently transfected with DDK tagged TFR1 cDNA. Lysate of transfected or non-transfected cells was immunoprecipitated with DDK or AF20 mAb, followed by sequential detection of the blot with DDK and TFR1 antibodies. **B**. Lysate of nontransfected Huh7 cells was incubated with AF20 mAb immobilized on protein G beads, or protein G beads alone to serve as a negative control, followed by Western blot detection of beads-associated proteins by antibodies against AF20, TFR1, HSP and N^+^/K^+^ ATPase, respectively. Note that a minigel was used for electrophoresis, which could not resolve proteins of similar sizes.

**Fig 4:**
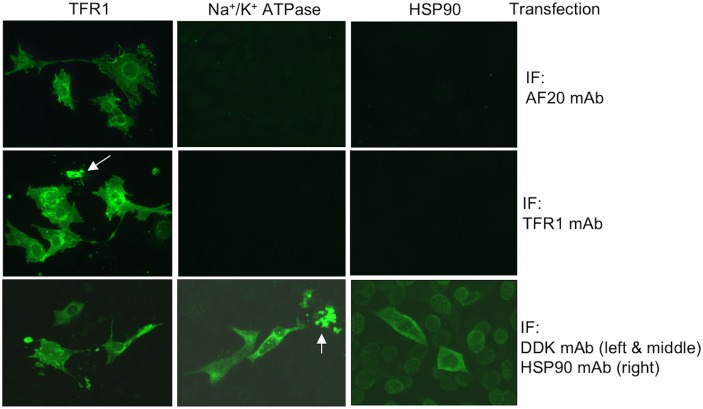
Affinity of AF20 mAb for TFR1 but not Na^+^/K^+^ ATPase or HSP90 by immunofluorescent staining. NIH 3T3 cells grown in 24-well plate with cover slips were transfected with expression constructs of DDK tagged TFR1, DDK tagged Na^+^/K^+^ ATPase, or untagged HSP90. Cells were fixed with acid alcohol followed by staining with AF20, TFR1, DDK, and HSP90 antibodies, respectively. Note that AF20 mAb only revealed signals in TFR1 transfected cells, although all three proteins were successfully expressed in NIH 3T3 cells. In addition, cell death or unusual morphology (elongated) was observed in cells expressing the exogenous proteins (dead cells are indicated by arrows). All images were taken at 20x magnification.

**Fig 5:**
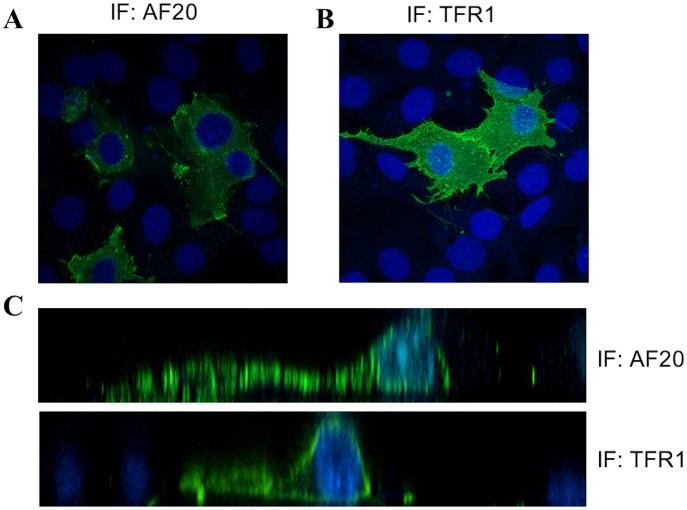
Subcellular localization of TFR1 as revealed by AF20 or TFR1 mAb. TFR1 transfected NIH 3T3 cells were fixed and stained with either AF20 mAb (panel A) or TFR1 mAb (panel B), followed by confocal microscopy to reveal subcellular localization and 3D pattern of the protein. (C) Stacks of X axis for panels A and B, respectively.

We reasoned that the AF20 antigen corresponded to a single host protein. The simultaneous purification of TFR1, HSP90, and ATPase by the AF20 mAb, as well as the presence of AF20 antigen in three concentrations of NaCl, was most likely a consequence of protein complex formation. To establish which one was the bona fide AF20 antigen, we performed *in vitro* reconstitution experiments using expression constructs for TFR1 (DDK tagged), Na^+^/K^+^ ATPase (DDK tagged) and HSP90 (untagged). DDK-tagged TFR1 expressed in Huh7 cells through transient transfection could be detected by IP—Western blot analysis using the DDK antibody as expected (Fig 3A, upper left panel). Interestingly, substituting the DDK antibody with AF20 antibody for IP did not compromise detection of the exogeneous TFR1 by the DDK antibody in Western blot (Fig 3A upper right panel). Reprobing the same blot with anti-TFR1 antibody revealed that endogenous TFR1 could be pulled down by the AF20 but not by the DDK antibody from non-transfected cells (Fig 3A, lower panel). In the non-transfected Huh7 cells, TFR1, HSP90, and ATPase could all be pulled down by the AF20 antibody, but not by the empty protein A/G beads (Fig 3B). In a different approach, we transfected cDNAs encoding DDK tagged TFR1 and Na^+^/K^+^ ATPase, or untagged HSP90 into NIH 3T3 cells, a cell line with little endogenous AF20 expression (Fig 1). Strong IF staining by AF20 antibody was documented in TFR1 but not Na^+^/K^+^ ATPase or HSP90 transfected NIH 3T3 cells (Fig 4), despite the fact that these two proteins were readily stained by DDK mAb or HSP90 antibody (Fig 4). Moreover, the AF20 antibody and TFR1 antibody revealed similar staining pattern in TFR1 transfected cells (Fig 4 left and Fig 5). These findings strongly implicated TFR1, rather than Na^+^/K^+^ ATPase or HSP90, as the AF20 antigen. Another interesting observation was that TFR1 transfected to NIH 3T3 cells exhibited not only cell surface localization, but also perinuclear staining as revealed by confocal microscopy (Figs 4 and 5). Moreover, from 2D or 3D view the staining by AF20 mAb and TFR1 mAb in TFR1 transfected cells was indistinguishable (Fig 5). We noticed that overexpression of the three proteins by transient transfection caused toxicity as indicated by morphological changes including cell shrinkage and elongation when compared with non-transfected cells (Fig 4 and data not shown).

### Holo but not apo transferrin could disrupt AF20 antibody binding to TFR1

**Fig 6:**
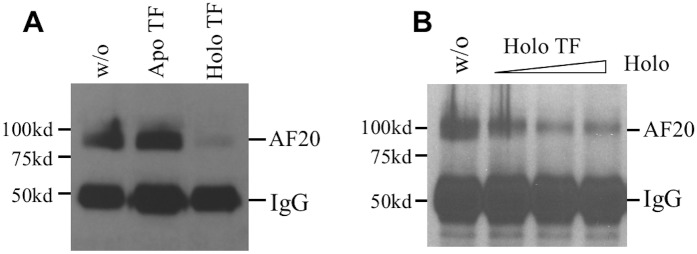
Holo but not apo transferrin could interfere with AF20 antigen-antibody interaction. **A**. Lysate of LS180 cells was subjected to immunoprecipitation with AF20 mAb in the absence (w/o) or presence of 33μg/ml of apo or holo transferrin (TF), and retained proteins were detected by Western blot with AF20 mAb. **B**. Increasing concentrations (3.3, 16.5, and 66μg/ml) of holo transferrin were added during immunoprecipitation with AF20 mAb, followed by Western blot with the same antibody.

Considering that diferric (holo) but not iron free (apo) transferrin is the ligand of TFR1 at neutral pH [5], we compared the two forms of transferrin for their impact on AF20 antigen—antibody interaction. Cell lysate was immunoprecipitated with AF20 antibody in the presence or absence of transferrin, followed by Western blot with AF20 antibody. We found that holo but not apo transferrin inhibited TFR1 precipitation by the AF20 antibody (Fig 6A). Increasing the concentration of holo transferrin from 3.3 to 66μg/ml caused a dose-dependent inhibition of the antibody—antigen interaction (Fig 6B). Note that further increase in the concentration of holo transferrin (≥ 66μg/ml) during immunoprecipitation did not further reduce the antigen-antibody reaction.

### Deglycosylation of the AF20 antigen abolished its affinity for AF20 antibody

**Fig 7:**
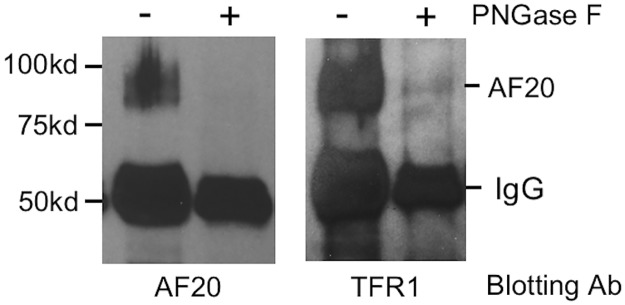
AF20 mAb failed to recognize deglycosylated TFR1. Huh7 cell lysate was subject to immunoprecipitation with AF20 mAb and retained proteins on protein G beads were treated with PNGase F at 37°C for 1hr. Samples were directly loaded onto 10% SDS-PAGE (minigel) in duplicate followed by Western blot detection using AF20 (left) and TFR1 antibodies (right), respectively. Note that an additional protein band above the 75-kd size marker was detected by TFR1 antibody from PNGase F treated sample, most likely corresponding to deglycosylated form of TFR1 (79kDd).

TFR1 contains three sites for N-linked glycosylation [6]. To determine whether N-linked glycosylation is essential for TFR1 recognition by the AF20 antibody, proteins pulled down by the AF20 antibody were treated with PNGase F. This enzyme cleaves between the innermost GLcNAc and asparagine residues of high mannose, hybrid, and complex oligosaccharides. It turned out that deglycosylated AF20 antigen could be no longer detected by the AF20 antibody in the Western blot (Fig 7, left panel), even after prolonged exposure (data not show). On the other hand, TFR1 antibody can detect both glycosylated and deglycosylated forms of TFR1, although the signal intensity was much reduced for the deglycosylated form (Fig 7, right panel). Alternatively, it is possible that deglycosylation makes TFR1 unstable with the intact protein dropped below the detection limit.

### Evaluation of TFR1 as a biomarker for early detection of malignant transformation

**Fig 8:**
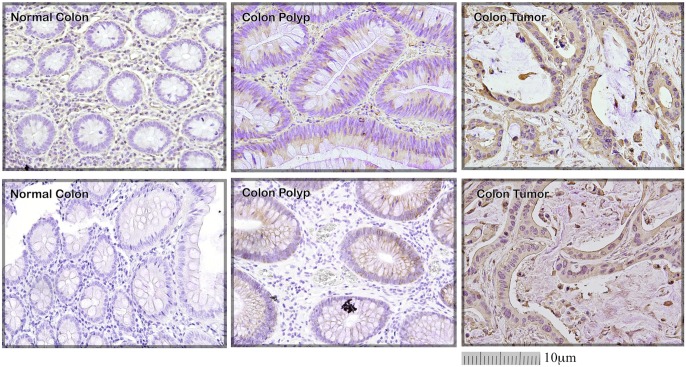
Correlation of AF20 expression with malignant transformation of colon tissues. A total of 4 pairs of tissue sections were stained with AF20 antibody followed by incubation with HRP-conjugated anti-mouse antibody. Two representative pairs are shown (upper and lower panels). Note that AF20 was clearly detectable in colon polyps (middle panels), and strongly expressed in colon cancer (right panels), but not in normal colon tissue (left panels). Images were taken at 20 x magnifications, with the scale bar provided.

**Fig 9:**
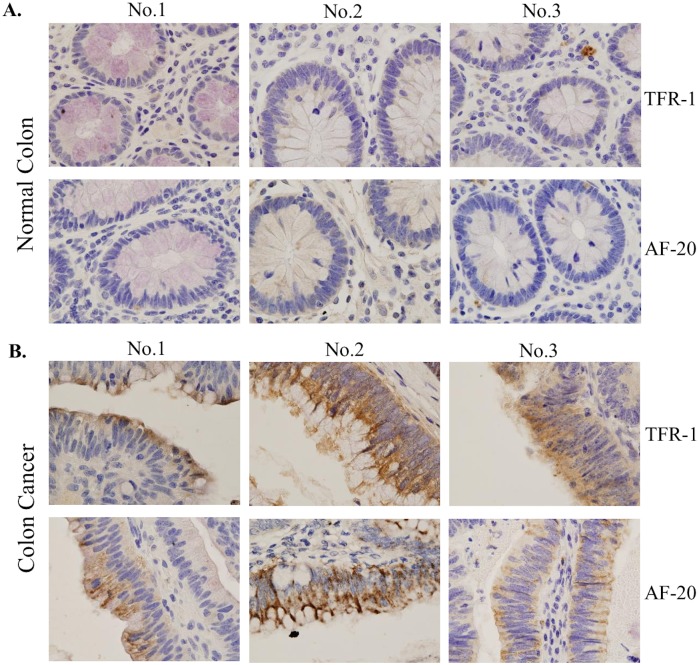
Correlation of TFR1 and AF20 staining in colon cancer samples. Three pairs of adjacent tissue sections of normal colon (**A**) and colon cancer (**B**) were stained with TFR1 (upper panels, 1:50 dilution) and AF20 mAb (lower panels, 1:500 dilution), respectively. Images were taken at 1000x magnification. Note that overexpression of both TFR1 and AF20 antigen was only observed in colon cancer but not in normal colon. In addition, the expression pattern and localization as revealed by TFR1 and AF20 Ab are indistinguishable.

AF20 antigen has been detected in majority of hepatoma and colon cancer cell lines [1]. To correlate AF20 expression with the stage of cancer development, we performed immunostaining in four paired samples including normal colon tissue, colon polyps, and colon cancer. While AF20 was undetectable in normal colon tissues, it was clearly detectable in polyps and strongly detected in colon cancer from all four pairs of samples (see Fig 8 for two representative pairs). In addition, relationship between AF20 and TFR1 was sought by their localization in colon cancer tissues. Three pairs of consecutive tissue sections from normal colon and colon cancer were stained with AF20 and TFR1 mAb, respectively. Both AF20 and TFR1 antibodies revealed strong signals in colon cancer with similar localization (Fig 9B). In contrast, no signal was detected from normal colon tissues by either antibody (Fig 9A). Together with immunostaining of TFR1 cDNA transfected cells by AF20 mAb (Fig 4), this finding strongly implicates TFR1 as the AF20 antigen.

## Discussion

Since the generation of the AF20 monoclonal antibody more than 20 years ago, the molecular identity of its target antigen has remained a longstanding puzzle. Extensive studies have revealed specific expression of AF20 antigen in human hepatoma and colon cancer cells. The rapid internalization of AF20 antibody in human hepatoma cell lines raised the possibility of specific and highly efficient treatment of liver cancer by conjugating small molecule drugs into the antibody [2,3]. While immunofluorescent staining clearly indicates its cell surface localization, detection by direct Western blot analysis has been unsuccessful. Rather, a combination of immunoprecipitation and Western blotting is needed. In the present study, we combined IP—SDS PAGE with ion-exchange chromatography to purify the AF20 antigen. Curiously, the AF20 antigen failed to be eluted from DEAE-cellulose column as a single peak. Rather, it was present in eluents of all the three NaCl concentrations suggesting variable affinities for the negatively charged column (Fig 2B). Peptide sequencing of the tryptic digests revealed two or three proteins instead of a single protein despite further purification by IP—SDS PAGE, which enriched AF20 antigen according to its immunological property as well as molecular size (90–110 kDa). Consequently, the three proteins identified: TFR1, HSP90, and Na^+^/K^+^ ATPase, share similar molecular weights. Indeed, in the subsequent reconstitution experiments using cDNA transfection, IP—Western blot analysis revealed ability of the AF20 antibody to immunoprecipitate TFR1, HSP90, as well as Na^+^/K^+^ ATPase. Considering that a monoclonal antibody recognizes an epitope of just 5–7 residues, the AF20 epitope could be present in all the three proteins. Alternatively and more likely, the AF20 epitope is present in just one of the three proteins described, and the other two proteins were co-purified with the true AF20 antigen through complex formation.

Two lines of experimental evidence supported the second interpretation and implicated TFR1, alternatively known as TFR, p90, and CD71 [7], as the bona fide AF20 antigen. First, transient transfection with TFR1 but not Na^+^/K^+^ ATPase or HSP90 cDNA conferred strong cell surface staining by AF20 antibody in NIH 3T3 cells, which express little endogenous AF20 antigen. Second, holo transferrin could compete for the AF20 antigen—antibody interaction during immunoprecipitation. As TFR1 has much greater affinity for the diferric transferrin [5], this finding also suggests the overlap between the transferrin binding site and the AF20 epitope. In addition, known features of TFR1 are also consistent with those of the AF20 antigen. For example, we previously identified AF20 antigen as a glycoprotein of 90 kDa which forms a homodimer of 180 kDa under nonreducing conditions [3]. This is exactly the case for TFR1, a dimeric glycoprotein with a monomeric size estimated at 90 or 95 kDa [8]. TFR2, on the other hand, is a protein of 355 residues (approximately 39 kDa). Moreover, ability of holo transferrin to interfere with the interaction of AF20 antigen—antibody is also compatible with TFR1, which has much greater affinity for diferric transferrin than TFR2.

TFR1 is a type II transmembrane protein. Its 760 residues consist of the short cytoplasmic domain (residues 1–67), a single transmembrane domain (residues 68–88), and a large extracellular domain containing three N-linked glycosylation sites. Tunicamycin treatment not only reduced size of TFR1 from 94 kDa to 79 kDa, but also prevented its dimerization. Interestingly, deglycosylated TFR1 lost affinity for transferrin [6]. Our finding that deglycosylated TFR1 is unable to bind AF20 antibody suggests a possible role of N-linked glycans in recognition by the AF20 mAb. Alternatively, deglycosylation of TFR1 renders the protein unstable to prevent binding by the AF20 mAb. TFR1 is responsible for iron deposition to most cell types. At neutral pH it has higher affinity for holo transferrin than apo transferrin. Following clathrin-mediated endocytosis, the acidic environment in the endosome triggers iron release from transferrin but increases the affinity of apo transferrin for TFR1 [5]. After fusion of endosomes with plasma membrane, the neutral pH promotes the release of apo transferrin from TFR1, thus completing the cycle of iron transport. A single cycle takes only 10–20 minutes [9], which is reminiscent of rapid internalization of the AF20 antibody in human hepatoma cells [3].

TFR1 is ubiquitously expressed in normal tissues at low level. Proliferating cells (such as fetal liver) have higher demand for iron than quiescent cells, which can explain their higher TFR1 expression [8]. The close association of TFR1 protein level with cell proliferation can also explain why it is overexpressed in many different types of tumors including adenocarcinoma [10], bladder transitional cell carcinoma [11], breast cancer [12], leukemia and lymphoma [13–15], pancreatic cancer [16], thyroid carcinoma [17], and tumor of the nervous system [18]. We previously found high expression of AF20 antigen in human hepatoma and colon cancer cell lines [1,3]. In the present study, AF20 was undetectable in normal colon but clearly detected in polyps and strongly positive in colon cancer (Figs 8 and 9). Consistent with our findings, TFR1 has been detected in hepatoma [19] and colorectal cancer [20,21]. More interestingly, TFR1 has been extensively studied as a delivery vehicle for anti-cancer drugs, either through its ligand, transferrin, or its monoclonal antibodies [7, 22–29]. Thus, our proposal to utilize AF20 mAb for the treatment of HCC is validated by findings from others [2–4].

In conclusion, identification of the extensively characterized AF20 antigen as glycosylated TFR1 explains nearly all the major features of AF20, including its homodimeric structure, cell surface localization, rapid endocytosis of antigen-antibody complex, overexpression in liver and colon cancers, and potential for cancer therapy. It also led to novel findings such as blocking of AF20 antigen-antibody interaction by diferric transferrin. In addition, our current study also revealed ability of TFR1 to form a complex with HSP90 and/or Na^+^/K^+^ ATPase or Mg^++^ ATPase. Whether TFR1 forms binary complex with HSP90 and ATPase, respectively or a tertiary complex remains to be determined. In this regard, the presence of AF20 antigen (TFR1) at eluents from 100mM, 200mM, and 400mM NaCl (Fig 2A) might reflect its different molecular forms (free form, HSP90 bound, ATPase bound, or tertiary complex). As shown in Fig 5C, overexpression of TFR1 alone led to its perinuclear staining in addition to cell surface localization. The biological, pathobiological, and therapeutic significance of TFR1 complex formation with HSP90 and/or ATPase warrants further investigation.
